# A close link between metabolic activity and functional connectivity in the resting human brain

**DOI:** 10.1186/2197-7364-2-S1-A78

**Published:** 2015-05-18

**Authors:** Susanne Passow, Karsten Specht, Tom Christian Adamsen, Martin Biermann, Njål Brekke, Alexander Richard Craven, Lars Ersland, Renate Grüner, Nina Kleven-Madsen, Ole-Heine Kvernenes, Thomas Schwarzlmüller, Rasmus Olesen, Kenneth Hugdahl

**Affiliations:** Department of Biological and Medical Psychology, University of Bergen, Norway; NORMENT Center of Excellence, University of Oslo, Norway; Department of Clinical Engineering, Haukeland University Hospital, Bergen, Norway; Department of Radiology, Haukeland University Hospital, Bergen, Norway; Department of Chemistry, University of Bergen, Norway; Department of Oncology and Medical Physics, Haukeland University Hospital, Bergen, Norway; Department of Physics and Technology, University of Bergen, Norway; Department of Clinical Medicine, University of Bergen, Norway; Center of Functionally Integrative Neuroscience and MINDLab, Aarhus University, Aarhus, Denmark; Division of Psychiatry, Haukeland University Hospital, Bergen, Norway

Default-mode network (DMN) functional connectivity and its task-dependent down-regulation have attracted a lot of attention in the field of neuroscience. Nevertheless, the exact underlying mechanisms of DMN functional connectivity, or more specifically, the blood oxygen level-dependent (BOLD) signal, are still not completely understood. To investigate more directly the association between local glucose consumption, local glutamatergic neurotransmission and DMN functional connectivity during rest, the present study combined for the first time 2-Deoxy-2-[18F]fluoroglucose positron emission tomography (FDG-PET), proton magnetic resonance spectroscopy (1H-MRS), and resting-state functional magnetic resonance imaging (rs-fMRI). Seed-based correlation analyses, using a key region of the DMN i.e. the dorsal posterior cingulate cortex as seed, revealed overall striking spatial similarities between fluctuations in FDG-uptake and the BOLD signal. More specifically, a conjunction analysis across both modalities showed that DMN areas as the inferior parietal lobe, angular gyrus, precuneus, middle and medial frontal gyrus were positively correlated with the dorsal posterior cingulate cortex. Furthermore, we could demonstrate that local glucose consumption in the medial frontal gyrus, posterior cingulate cortex and left angular gyrus was associated with functional connectivity within the DMN. We did not find a relationship between glutamatergic neurotransmission and functional connectivity. In line with very recent findings, our results provide further evidence for a close association between local metabolic activity and functional connectivity and enable further insights towards a better understanding of the underlying mechanisms of the BOLD signal.

